# Substantial health and economic burden of COVID-19 during the year after acute illness among US adults not at high risk of severe COVID-19

**DOI:** 10.1186/s12916-023-03235-5

**Published:** 2024-02-02

**Authors:** Amie Scott, Wajeeha Ansari, Richard Chambers, Maya Reimbaeva, Tomasz Mikolajczyk, Michael Benigno, Florin Draica, Joanna Atkinson

**Affiliations:** 1 Global Real World Evidence, Pfizer Inc, 235 East 42nd Street, New York, NY 10017 USA; 2 Global HEOR, Pfizer Inc, New York, NY USA; 3 Global Product Development Statistics, Pfizer Inc, New York, NY USA; 4 Global Biometrics and Data Management, Pfizer Inc, Groton, CT USA; 5 Quanticate, Warsaw, Poland; 6 Medical Affairs, Pfizer Inc, New York, NY USA; 7 Medical Affairs, Pfizer Ltd, Tadworth, Surrey, UK

**Keywords:** COVID-19, SARS-CoV-2, Long COVID, PASC, Post-COVID conditions

## Abstract

**Background:**

Patients recovering from SARS-CoV-2 infection and acute COVID-19 illness can experience a range of long-term post-acute effects. The potential clinical and economic burden of these outcomes in the USA is unclear. We evaluated diagnoses, medications, healthcare utilization, and medical costs before and after acute COVID-19 illness in US patients who were not at high risk of severe COVID-19.

**Methods:**

This study included eligible adults who were diagnosed with COVID-19 from April 1 to May 31, 2020, who were 18 − 64 years of age, and enrolled within Optum’s de-identified Clinformatics® Data Mart Database for 12 months before and 13 months after COVID-19 diagnosis. Patients with any condition or risk factor placing them at high risk of progression to severe COVID-19 were excluded. Percentages of diagnoses, medications, healthcare utilization, and costs were calculated during baseline (12 months preceding diagnosis) and the post-acute phase (12 months after the 30-day acute phase of COVID-19). Data were stratified into 3 cohorts according to disposition during acute COVID-19 illness (i.e., not hospitalized, hospitalized without intensive care unit [ICU] admission, or admitted to the ICU).

**Results:**

The study included 3792 patients; 56.5% of patients were men, 44% were White, and 94% did not require hospitalization. Compared with baseline, patients during the post-acute phase had percentage increases in the diagnosis of the following disorders: blood (166%), endocrine and metabolic (123%), nervous system (115%), digestive system (76%), and mental and behavioral (75%), along with increases in related prescriptions. Substantial increases in all measures of healthcare utilization were observed among all 3 cohorts. Total medical costs increased by 178% during the post-acute phase. Those who were hospitalized with or without ICU admission during the acute phase had the greatest increases in comorbidities and healthcare resource utilization. However, the burden was apparent across all cohorts.

**Conclusions:**

As evidenced by resource use in the post-acute phase, COVID-19 places a significant long-term clinical and economic burden among US individuals, even among patients whose acute infection did not merit hospitalization.

**Supplementary Information:**

The online version contains supplementary material available at 10.1186/s12916-023-03235-5.

## Background

Nearly 20% of adults diagnosed with COVID-19 experience symptoms for ≥ 3 months after first contracting the virus [1, 2]. These highly variable signs and symptoms, often termed post-COVID conditions, can either begin at the time of initial infection and persist for several months or may be new symptoms or syndromes that develop only after the acute phase of COVID-19 [3–5]. For many individuals, post-COVID conditions involve multiple organ systems and significantly impair daily functioning and productivity [6]. Although a universally accepted definition and timeframe of the condition has not yet been developed, a clinical diagnosis of post-acute sequelae of COVID-19 (PASC) was assigned an International Classification of Diseases 10 (ICD-10) code (U09.9) in October of 2021 [7, 8].

Although it is widely accepted that older age, belonging to racial and ethnic minority groups, and certain underlying medical conditions are associated with an increased risk of progression to severe COVID-19 upon initial infection [9], the characteristics associated with risk of developing post-COVID conditions are largely unknown. Some overlapping but distinct risk factors, such as female sex and older age, have been identified, as well as an association between acute COVID-19 severity and duration of symptoms [10–12]. However, numerous reports from several countries have identified high rates of post-COVID conditions, even among patient cohorts with mixed disease severity or mild cases of COVID-19 [4, 13–15]. In the current landscape where mild COVID-19 illness is becoming more common, owing both to vaccination [16] and the highly transmissible but potentially less virulent Omicron strain [16–19], it is necessary to understand the burden on health and healthcare systems after an acute COVID-19 infection among patients who do not have underlying comorbidities and who did not require hospitalization for acute COVID-19. An understanding of long-term health effects after acute COVID-19 infection, the populations at risk, and the associated strain on healthcare systems is imperative to inform accurate estimations of the evolving clinical and economic burden of COVID-19.

We conducted a descriptive, retrospective analysis of morbidity, healthcare resource utilization, and costs associated with the post-acute phase of COVID-19 among adult patients aged < 65 years and without any underlying conditions placing them at high risk of progression to severe disease. In a companion report [20], we describe an identical analysis conducted among patients with ≥ 1 underlying condition or who were aged ≥ 65 years. The companion report and the current report were both purely descriptive with no formal statistical comparisons, with the goal to present a broad and unbiased dataset that can inform future hypothesis generation.

## Methods

This descriptive, retrospective cohort study compared baseline healthcare utilization data from patients during the year before contracting COVID-19 with their healthcare utilization data during the year after recovery from the 30-day acute phase (from day 31 through day 390 after diagnosis). All patients served as their own control for evaluation of diagnoses, medications, healthcare utilization, and costs before compared with after acute COVID-19. Details regarding the study design, data source, and inclusion and exclusion criteria have been included in the companion manuscript regarding individuals at high risk of progression to severe COVID-19 [20].

Briefly, enrolled patients were diagnosed with COVID-19 (ICD-10 diagnosis code of U07.1) between April 1 and May 31, 2020 (the index period). Information regarding diagnoses, medications, healthcare utilization, and costs were collected from Optum’s de-identified Clinformatics® Data Mart Database (CDM), which contains patient-level information derived from administrative health claims of commercial and Medicare Advantage plan members. Data extracted from CDM were not sufficient to determine whether any post-COVID-19 diagnosis, medication prescription, or healthcare utilization was specifically caused by COVID-19; therefore, it was unknown whether any individual diagnosis or adverse health outcome was truly a “post-COVID condition” or was caused by unrelated factors [5]. Eligible patients were continuously enrolled in CDM (with gaps of ≤ 45 days permitted) over the 12 months before and 13 months after COVID-19 diagnosis and were aged 18 to 64 years on the index date. Patients were excluded if they had a diagnosis code (ICD-10-Clinical Modification [ICD-10-CM]), procedure code (ICD-10-Procedure Coding System [ICD-10-PCS], Current Procedural Terminology [CPT®], Healthcare Common Procedures Coding System [HCPCS]), or National Drug Code (NDC) for any condition placing them at high risk of progression to severe COVID-19, per CDC definitions as of October 14, 2021 [9], within the 12 months before the index date. Additional exclusion criteria were hospitalizations for ≥ 5 consecutive days during the baseline phase; any time spent at a long-term care facility, skilled nursing facility, inpatient rehabilitation, or hospice during baseline or at index date; an ICD-10 code for confirmed COVID-19 before the index period; or death during the acute phase of COVID-19.

The top 500 ICD-10 diagnosis codes were analyzed by ICD-10 code chapter (a system of organization based on the most affected organ systems or types of injury/disease), and medications were categorized according to Uniform System of Classification class. All diagnosis and medication categories applicable to < 2% of the overall population during the baseline phase were excluded from analysis. The “biologics” category was also excluded based on incomplete data capture. Standard medical cost means, standard deviations, medians, and quartiles were calculated using the number of patients with a related visit or service, and nonzero costs were calculated using the number of patients with a cost > 0 associated with that visit or service. No adjustments were made for patients who died during the 12-month post-acute phase; all deaths that occurred during the post-acute phase were accounted for and reported. Absolute and relative change from baseline to the post-acute phase were calculated for each outcome where possible using frequency counts. To better understand the relationship between post-acute outcomes and initial infection severity, all data were presented for the overall population and stratified by patient disposition during the acute phase of COVID-19 (within 30 days after diagnosis): not hospitalized, hospitalized without intensive care unit (ICU) admission, or admitted to the ICU. Analyses were performed using SAS version 9.4 (SAS, Cary, NC), and no statistical inference tests were conducted.

## Results

### Patient population

Overall, the cohort included 3792 patients with a median (quartile 1; quartile 3) age of 40 (31; 50) years (Table 1). A slight majority of patients were male (56.5%), and 44.0% of patients were White. When categorized by disposition during the acute phase of COVID-19, 3546 patients (93.5%) did not require hospitalization, 164 (4.3%) were hospitalized without ICU admission, and 82 (2.2%) were admitted to the ICU. Patients who were male, Black, or aged 50 to 64 years were more highly represented within the cohort with ICU admission than within the overall population.

**Table 1:** Baseline demographic and clinical characteristics

		**Disposition during acute COVID-19 illness**		
**Characteristic**	**All patients** **(*N* = 3792)**	**No hospitalization** **(*n* = 3546)**	**Hospitalization without ICU admission** **(*n* = 164)**	**ICU admission** **(*n* = 82)**
Sex, *n* (%)				
Female	1648 (43.5)	1571 (44.3)	56 (34.1)	21 (25.6)
Male	2144 (56.5)	1975 (55.7)	108 (65.9)	61 (74.4)
Age, years				
Mean ± SD	40.4 ± 12.2	39.9 ± 12.2	46.4 ± 11.8	48.0 ± 10.3
Median (Q1; Q3)	40 (31; 50)	39 (30; 50)	47 (36.5; 56)	50.5 (41; 56)
Age group, years, *n* (%)				
18–29	859 (22.7)	838 (23.6)	15 (9.1)	6 (7.3)
30–49	1900 (50.1)	1791 (50.5)	78 (47.6)	31 (37.8)
50–64	1033 (27.2)	917 (25.9)	71 (43.3)	45 (54.9)
Race or ethnicity, *n* (%)				
White	1670 (44.0)	1599 (45.1)	49 (29.9)	22 (26.8)
Black	397 (10.5)	359 (10.1)	21 (12.8)	17 (20.7)
Hispanic	1078 (28.4)	984 (27.7)	66 (40.2)	28 (34.1)
Asian	229 (6.0)	215 (6.1)	10 (6.1)	4 (4.9)
Unknown	418 (11.0)	389 (11.0)	18 (11.0)	11 (13.4)
Geographic division, *n* (%)				
New England	236 (6.2)	227 (6.4)	7 (4.3)	2 (2.4)
Mid-Atlantic	764 (20.1)	730 (20.6)	27 (16.5)	7 (8.5)
East North Central	729 (19.2)	670 (18.9)	39 (23.8)	20 (24.4)
West North Central	402 (10.6)	374 (10.5)	20 (12.2)	8 (9.8)
South Atlantic	664 (17.5)	617 (17.4)	33 (20.1)	14 (17.1)
East South Central	93 (2.5)	87 (2.5)	4 (2.4)	2 (2.4)
West South Central	330 (8.7)	305 (8.6)	14 (8.5)	11 (13.4)
Mountain	295 (7.8)	285 (8.0)	5 (3.0)	5 (6.1)
Pacific	252 (6.7)	236 (6.7)	10 (6.1)	6 (7.3)
Insurance, *n* (%)				
Commercial	3756 (99.1)	3523 (99.4)	155 (94.5)	78 (95.1)
Medicare	36 (0.9)	23 (0.6)	9 (5.5)	4 (4.9)
Care setting of COVID-19 diagnosis, *n* (%)				
Inpatient	181 (4.8)	0 (0.0)	128 (78.0)	53 (64.6)
Outpatient	3611 (95.2)	3546 (100)	36 (22.0)	29 (35.4)
Disposition during acute COVID-19 illness, *n* (%)				
No hospitalization	3546 (93.5)			
Hospitalization without ICU admission	164 (4.3)			
ICU admission	82 (2.2)			
COVID-19 inpatient stay that overlaps acute and post-acute phases (> 30 days), *n* (%)	9 (0.2)	0 (0.0)	2 (1.2)	7 (8.5)

*ICU* intensive care unit, *Q1* quartile 1, *Q3* quartile 3

Most patients (95.2%) were diagnosed with COVID-19 in the outpatient setting (Table 1). Very few patients (0.2% of those hospitalized at any time during the acute phase) had hospital stays that lasted > 30 days, overlapping the acute and post-acute phases of the study. None of the patients died during the post-acute phase. A full list of reasons for exclusion from the analysis is shown in Table S1.

### Diagnoses

**Fig. 1:**
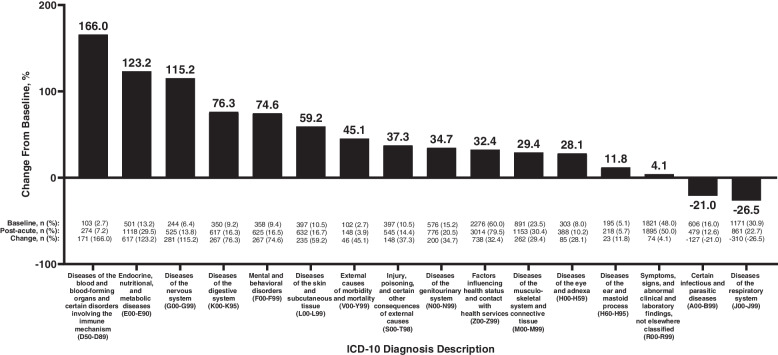
Percentage change from baseline to post-acute phase in ICD-10 diagnoses in the overall population (*N* = 3792). Chapters include those with a prevalence of ≥ 2% in the baseline population * ICD-10* International Classification of Diseases 10th Revision

In the overall population, the frequency of ICD-10 diagnosis codes increased within several chapters (diagnosis categories) between the baseline and post-acute phases (Fig. 1). The number of patients with “diseases of the blood and blood-forming organs and certain disorders involving the immune mechanism” increased by 166.0% from baseline, as well as a 123.2% increase in “endocrine, nutritional, and metabolic diseases,” a 115.2% increase in “diseases of the nervous system,” a 76.3% increase in “diseases of the digestive system,” and a 74.6% increase in “mental and behavioral disorders” (Fig. 1). Frequencies of diagnoses within these chapters increased within all 3 cohorts (Table 2), with the smallest percentage increases observed among patients who were not hospitalized for acute COVID-19 and the largest percentage increases observed among patients who were admitted to the ICU.

**Table 2:** Diagnoses during the baseline and post-acute phases^a^ stratified by disposition during acute COVID-19

		**No hospitalization** **(*n* = 3546)**			**Hospitalization without ICU admission** **(*n* = 164)**			**ICU admission** **(*n* = 82)**		
**ICD diagnosis description**	**ICD diagnosis code**	**Baseline phase, *n* (%)**	**Post-acute phase, *n* (%)**	**Change from baseline to post-acute phase,** **Δ (% change)**	**Baseline phase, *n* (%)**	**Post-acute phase, *n* (%)**	**Change from baseline to post-acute phase,** **Δ (% change)**	**Baseline phase, *n* (%)**	**Post-acute phase, *n* (%)**	**Change from baseline to post-acute phase,** **Δ (% change)**
Diseases of the blood and blood-forming organs and certain disorders involving the immune mechanism	D50-D89	96 (2.7)	242 (6.8)	146 (152.1)	6 (3.7)	20 (12.2)	14 (233.3)	1 (1.2)	12 (14.6)	11 (1100.0)
Endocrine, nutritional, and metabolic diseases	E00-E90	476 (13.4)	1008 (28.4)	532 (111.8)	19 (11.6)	67 (40.9)	48 (252.6)	6 (7.3)	43 (52.4)	37 (616.7)
Diseases of the nervous system	G00-G99	231 (6.5)	477 (13.5)	246 (106.5)	10 (6.1)	27 (16.5)	17 (170.0)	3 (3.7)	21 (25.6)	18 (600.0)
Diseases of the digestive system	K00-K95	326 (9.2)	560 (15.8)	234 (71.8)	21 (12.8)	39 (23.8)	18 (85.7)	3 (3.7)	18 (22.0)	15 (500.0)
Mental and behavioral disorders	F00-F99	349 (9.8)	582 (16.4)	233 (66.8)	7 (4.3)	23 (14.0)	16 (228.6)	2 (2.4)	20 (24.4)	18 (900.0)
Diseases of the skin and subcutaneous tissue	L00-L99	383 (10.8)	585 (16.5)	202 (52.7)	12 (7.3)	31 (18.9)	19 (158.3)	2 (2.4)	16 (19.5)	14 (700.0)
External causes of morbidity and mortality	V00-Y99	96 (2.7)	137 (3.9)	41 (42.7)	5 (3.0)	9 (5.5)	4 (80.0)	1 (1.2)	2 (2.4)	1 (100.0)
Injury, poisoning, and certain other consequences of external causes	S00-T98	379 (10.7)	503 (14.2)	124 (32.7)	14 (8.5)	32 (19.5)	18 (128.6)	4 (4.9)	10 (12.2)	6 (150.0)
Diseases of the genitourinary system	N00-N99	558 (15.7)	726 (20.5)	168 (30.1)	16 (9.8)	27 (16.5)	11 (68.8)	2 (2.4)	23 (28.0)	21 (1050.0)
Factors influencing health status and contact with health services	Z00-Z99	2166 (61.1)	2834 (79.9)	668 (30.8)	79 (48.2)	120 (73.2)	41 (51.9)	31 (37.8)	60 (73.2)	29 (93.5)
Diseases of the musculoskeletal system and connective tissue	M00-M99	843 (23.8)	1063 (30.0)	220 (26.1)	37 (22.6)	59 (36.0)	22 (59.5)	11 (13.4)	31 (37.8)	20 (181.8)
Diseases of the eye and adnexa	H00-H59	280 (7.9)	354 (10.0)	74 (26.4)	16 (9.8)	27 (16.5)	11 (68.8)	7 (8.5)	7 (8.5)	0 (0.0)
Diseases of the ear and mastoid process	H60-H95	184 (5.2)	206 (5.8)	22 (12.0)	7 (4.3)	9 (5.5)	2 (28.6)	4 (4.9)	3 (3.7)	–1 (–25.0)
Symptoms, signs, and abnormal clinical laboratory findings not elsewhere classified	R00-R99	1715 (48.4)	1735 (48.9)	20 (1.2)	75 (45.7)	104 (63.4)	29 (38.7)	31 (37.8)	56 (68.3)	25 (80.6)
Certain infectious and parasitic diseases	A00-B99	574 (16.2)	430 (12.1)	–144 (–25.1)	24 (14.6)	26 (15.9)	2 (8.3)	8 (9.8)	23 (28.0)	15 (187.5)
Diseases of the respiratory system	J00-J99	1107 (31.2)	764 (21.5)	–343 (–31.0)	42 (25.6)	60 (36.6)	18 (42.9)	22 (26.8)	37 (45.1)	15 (68.2)

*ICD* International Classification of Diseases, *ICU* intensive care unit

^a^The baseline period was the 12 months before the index date, and the post-acute phase spanned from 1 to 13 months after the index date

**Fig. 2:**
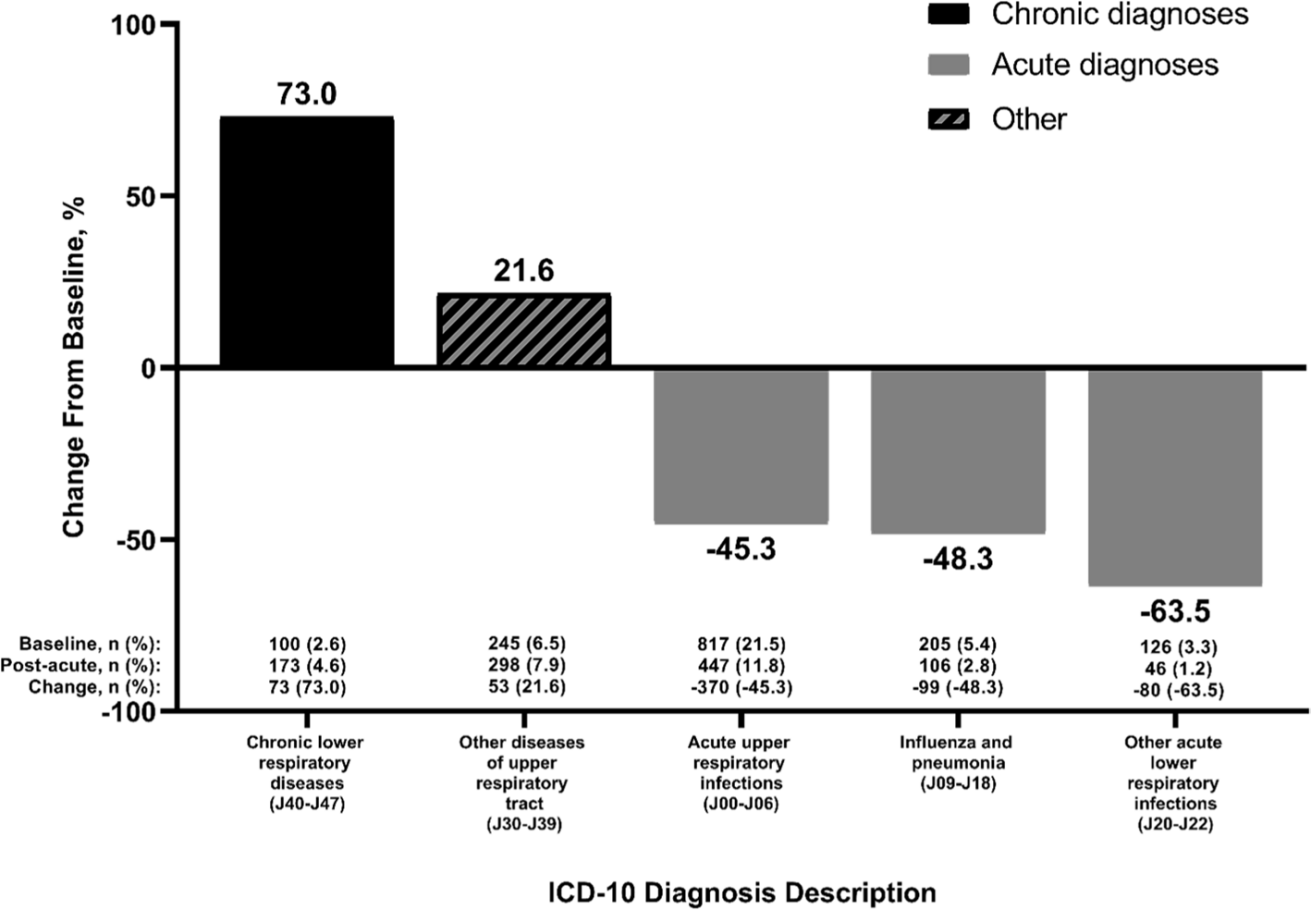
Diagnoses of the respiratory system during baseline and post-acute phases in the overall population (*N* = 3792). Diagnoses include those with a prevalence of ≥ 2% in the baseline population and are categorized according to whether they are chronic, acute, or other/both * ICD-10* International Classification of Diseases 10th Revision

The greatest decrease observed within the overall cohort was a − 26.5% change from baseline in the frequency of “diseases of the respiratory system” (Fig. 1). This value reflected a combination of decreased acute diagnoses, as well as a 73.0% increase in the frequency of “chronic lower respiratory diseases” (Fig. 2).

### Medication use

In the overall population, medications from several different classes were prescribed in greater numbers during the post-acute phase compared with baseline (Table 3). The greatest observed changes were a 188.6% increase in hormones (primarily corticoids and glucocorticoids; Table S2), a 113.1% increase in vascular agents, a 62.7% increase in musculoskeletal agents, a 61.5% increase in antihyperlipidemic agents, and a 61.0% increase in neurological/neuromuscular agents. In contrast, there was also a 73.5% decrease in prescriptions of cough/cold/flu preparations and a 48.8% decrease in antivirals.

**Table 3:** New medication prescriptions during baseline and post-acute phases^a^ in the overall population (*N* = 3792)

USC medication class description	Baseline phase, *n* (%)	Post-acute phase, *n* (%)	Change from baseline to post-acute phase, Δ (% change)
Hormones	228 (6.0)	658 (17.4)	430 (188.6)
Vascular agents	130 (3.4)	277 (7.3)	147 (113.1)
Musculoskeletal	153 (4.0)	249 (6.6)	96 (62.7)
Antihyperlipidemic agents	148 (3.9)	239 (6.3)	91 (61.5)
Neurological/neuromuscular disorders	118 (3.1)	190 (5.1)	72 (61.0)
Analgesics	302 (8.0)	485 (12.8)	183 (60.6)
Psychotherapeutic drugs	354 (9.3)	560 (14.8)	206 (58.2)
Gastrointestinal	191 (5.0)	285 (7.5)	94 (49.2)
Ophthalmic preparations	112 (3.0)	157 (4.1)	45 (40.2)
Genitourinary	168 (4.4)	235 (6.2)	67 (39.9)
Antinauseants	162 (4.3)	213 (5.6)	51 (31.5)
Antiarthritics	381 (10.0)	492 (13.0)	111 (29.1)
Dermatologicals	139 (3.7)	171 (4.5)	32 (23.0)
Thyroid therapy	105 (2.8)	129 (3.4)	24 (22.9)
Anti-fungal agents	206 (5.4)	236 (6.2)	30 (14.6)
Contraceptives	278 (7.3)	281 (7.4)	3 (1.1)
Anti-infectives, systemic	1020 (26.9)	928 (24.5)	–92 (–9.0)
Respiratory therapy	469 (12.4)	369 (9.7)	–100 (–21.3)
Antivirals	287 (7.6)	147 (3.9)	–140 (–48.8)
Cough/cold/flu preparations	302 (8.0)	80 (2.1)	–222 (–73.5)

*USC* Uniform System of Classification

^a^The baseline period was the 12 months before the index date, and the post-acute phase spanned from 1 to 13 months after the index date

For most medication classes, increases in prescriptions from the baseline to the post-acute phase were observed across all 3 cohorts but were generally greatest among those who were admitted to the ICU for acute COVID-19 (Table 4). As an exception, the magnitude of the percentage increases in hormone prescriptions was relatively consistent across cohorts.

**Table 4:** New medication prescriptions during the baseline and post-acute phases^a^ stratified by disposition during acute COVID-19

USC medication class description	No hospitalization (*n* = 3546)			Hospitalization without ICU admission (*n* = 164)			ICU admission (*n* = 82)		
	**Baseline phase, *n* (%)**	**Post-acute phase, *n* (%)**	**Change from baseline to post-acute phase, Δ (% change)**	**Baseline phase, *n* (%)**	**Post-acute phase, *n* (%)**	**Change from baseline to post-acute phase,** **Δ (% change)**	**Baseline phase, *n* (%)**	**Post-acute phase, *n* (%)**	**Change from baseline to post-acute phase, Δ (% change)**
Hormones	214 (6.0)	617 (17.4)	403 (188.3)	9 (5.5)	27 (16.5)	18 (200.0)	5 (6.1)	14 (17.1)	9 (180.0)
Vascular agents	114 (3.2)	227 (6.4)	113 (99.1)	9 (5.5)	25 (15.2)	16 (177.8)	7 (8.5)	25 (30.5)	18 (257.1)
Musculoskeletal	140 (3.9)	232 (6.5)	92 (65.7)	11 (6.7)	12 (7.3)	1 (9.1)	2 (2.4)	5 (6.1)	3 (150.0)
Antihyperlipidemic agents	136 (3.8)	208 (5.9)	72 (52.9)	8 (4.9)	17 (10.4)	9 (112.5)	4 (4.9)	14 (17.1)	10 (250.0)
Neurological/neuromuscular disorders	110 (3.1)	171 (4.8)	61 (55.5)	5 (3.0)	10 (6.1)	5 (100.0)	3 (3.7)	9 (11.0)	6 (200.0)
Analgesics	274 (7.7)	438 (12.4)	164 (59.9)	17 (10.4)	29 (17.7)	12 (70.6)	11 (13.4)	18 (22.0)	7 (63.6)
Psychotherapeutic drugs	341 (9.6)	520 (14.7)	179 (52.5)	9 (5.5)	24 (14.6)	15 (166.7)	4 (4.9)	16 (19.5)	12 (300.0)
Gastrointestinal	178 (5.0)	258 (7.3)	80 (44.9)	12 (7.3)	19 (11.6)	7 (58.3)	1 (1.2)	8 (9.8)	7 (700.0)
Ophthalmic preparations	102 (2.9)	143 (4.0)	41 (40.2)	8 (4.9)	12 (7.3)	4 (50.0)	2 (2.4)	2 (2.4)	0 (0.0)
Genitourinary	161 (4.5)	222 (6.3)	61 (37.9)	7 (4.3)	6 (3.7)	–1 (–14.3)	0 (0.0)	7 (8.5)	7 (NC)
Antinauseants	149 (4.2)	194 (5.5)	45 (30.2)	10 (6.1)	13 (7.9)	3 (30.0)	3 (3.7)	6 (7.3)	3 (100.0)
Antiarthritics	345 (9.7)	452 (12.7)	107 (31.0)	25 (15.2)	26 (15.9)	1 (4.0)	11 (13.4)	14 (17.1)	3 (27.3)
Dermatologicals	135 (3.8)	160 (4.5)	25 (18.5)	2 (1.2)	3 (1.8)	1 (50.0)	2 (2.4)	8 (9.8)	6 (300.0)
Thyroid therapy	103 (2.9)	123 (3.5)	20 (19.4)	1 (0.6)	3 (1.8)	2 (200.0)	1 (1.2)	3 (3.7)	2 (200.0)
Anti-fungal agents	197 (5.6)	221 (6.2)	24 (12.2)	6 (3.7)	8 (4.9)	2 (33.3)	3 (3.7)	7 (8.5)	4 (133.3)
Contraceptives	273 (7.7)	273 (7.7)	0 (0.0)	3 (1.8)	6 (3.7)	3 (100.0)	2 (2.4)	2 (2.4)	0 (0.0)
Anti-infectives, systemic	963 (27.2)	867 (24.5)	–96 (–10.0)	39 (23.8)	43 (26.2)	4 (10.3)	18 (22.0)	18 (22.0)	0 (0.0)
Respiratory therapy	442 (12.5)	328 (9.3)	–114 (–25.8)	18 (11.0)	25 (15.2)	7 (38.9)	9 (11.0)	16 (19.5)	7 (77.8)
Antivirals	280 (7.9)	142 (4.0)	–138 (–49.3)	2 (1.2)	4 (2.4)	2 (100.0)	5 (6.1)	1 (1.2)	–4 (–80.0)
Cough/cold/flu preparations	282 (8.0)	70 (2.0)	–212 (–75.2)	14 (8.5)	6 (3.7)	–8 (–57.1)	6 (7.3)	4 (4.9)	–2 (–33.3)

*ICU* intensive care unit, *NC* not calculable, *USC* Uniform System of Classification

^a^The baseline period was the 12 months before the index date, and the post-acute phase spanned from 1 to 13 months after the index date

Prescription increases were also observed within several medication classes that were not included in the main analysis because they were prescribed to < 2% of the overall cohort during the baseline phase (Table S3). These included increases of > 100% within the classes of hemostatic modifiers, diabetes therapy, cardiac agents, and blood factors.

### Medical care and hospitalizations

Between the baseline and post-acute phases, increases were observed across all measures of healthcare utilization in the overall population and among all 3 cohorts (Tables 5 and 6). The greatest changes were related to inpatient resources: a 3100% increase in inpatient lab tests, a 3000% increase in total days spent in the ICU, a 1269% increase in length of stay (LOS), and a 527% increase in number of hospitalizations (Table 5). With the exception of emergency department (ED) visits, increases in all measures of healthcare utilization were greatest among those who were admitted to the ICU for acute COVID-19 (Table 6).

**Table 5:** Healthcare resource utilization during the baseline and post-acute phases^a^ in the overall population (*N* = 3792)

Visit or procedure	Baseline phase, *n* (%)	Post-acute phase, *n* (%)	Change from baseline to post-acute phase, Δ (% change)
Outpatient lab tests			
Tests	2200	4750	2550 (115.9)
Patients	1253	1768	515 (41.1)
Mean ± SD	0.6 ± 1.1	1.3 ± 2.6	0.7
Median (Q1; Q3)	0 (0; 1)	0 (0; 2)	0
Outpatient visits (specialist or nonspecialist)			
Visits	18,691	39,570	20,879 (111.7)
Patients	3026	3316	290 (9.6)
Mean ± SD	4.9 ± 6.7	10.4 ± 14.0	5.5
Median (Q1; Q3)	3 (1; 6)	6 (2; 13)	3
Emergency department visits			
Visits	492	662	170 (34.6)
Patients	392	471	79 (20.2)
Mean ± SD	0.1 ± 0.4	0.2 ± 0.6	0.0
Median (Q1; Q3)	0 (0; 0)	0 (0; 0)	0
Prescription classes			
Prescriptions	5713	7539	1826 (32.0)
Patients	2255	2541	286 (12.7)
Mean ± SD	1.5 ± 1.8	2.0 ± 2.2	0.5
Median (Q1; Q3)	1 (0; 2)	1 (0; 3)	0
Inpatient visits			
Visits	22	138	116 (527.3)
Patients	21	94	73 (347.6)
Mean ± SD	0.0 ± 0.1	0.0 ± 0.3	0.0
Median (Q1; Q3)	0 (0; 0)	0 (0; 0)	0
Inpatient lab tests			
Tests	1	32	31 (3100)
Patients	1	11	10 (1000.0)
Mean ± SD	0.0 ± 0.0	0.0 ± 0.2	0.0
Median (Q1; Q3)	0 (0; 0)	0 (0; 0)	0
Length of hospital stay			
Days	86	1177	1091 (1268.6)
Patients	21	94	73 (347.6)
Mean ± SD	4.1 ± 3.8	12.5 ± 25.8	8.4 (205.8)
Median (Q1; Q3)	3 (2; 5)	4 (3; 8)	1
Length of ICU stay			
Days	1	31	30 (3000.0)
Patients	1	17	16 (1600.0)
Mean ± SD	0.0 ± 0.2	0.3 ± 1.1	0.3 (592.6)
Median (Q1; Q3)	0 (0; 0)	0 (0; 0)	0
Invasive mechanical ventilation use, patient *n* (%)	0 (0.0)	5 (0.1)	5 (NC)
Noninvasive mechanical ventilation use, patient *n* (%)	0 (0.0)	4 (0.1)	4 (NC)
Supplemental oxygen use, patient *n* (%)	0 (0.0)	7 (0.2)	7 (NC)
Readmission within 30 days, patient *n* (%)	0 (0.0)	23 (0.6)	23 (NC)

*ICU* intensive care unit, *NC* not calculable, *Q1* quartile 1, *Q3* quartile 3

For visits, tests, prescriptions, and procedures, means or percentages were calculated using the total number of patients within the cohort as the denominator. For length of hospital stay and ICU stay, means were calculated as the total number of days divided by the number of patients with any inpatient hospital stay

^a^The baseline period was the 12 months before the index date, and the post-acute phase spanned from 1 to 13 months after the index date

**Table 6:** Healthcare resource utilization during the baseline and post-acute phases^a^ stratified by disposition during acute COVID-19

Visit or procedure	No hospitalization (*n* = 3546)			Hospitalization without ICU admission (*n* = 164)			ICU admission (*n* = 82)		
	**Baseline phase**	**Post-acute phase**	**Change from baseline to post-acute phase,** **Δ (% change)**	**Baseline phase**	**Post-acute phase**	**Change from baseline to post-acute phase,** **Δ (% change)**	**Baseline phase**	**Post-acute phase**	**Change from baseline to post-acute phase,** **Δ (% change)**
Outpatient lab tests									
Tests, *n*	2121	4518	2397 (113.0)	60	156	96 (160.0)	19	76	57 (300.0)
Patients, *n*	1204	1662	458 (38.0)	37	71	34 (91.9)	12	35	23 (191.7)
Mean ± SD	0.6 ± 1.1	1.3 ± 2.6	0.7	0.4 ± 0.9	1.0 ± 1.9	0.6	0.2 ± 0.7	0.9 ± 1.4	0.7
Median (Q1; Q3)	0 (0; 1)	0 (0; 2)	0	0 (0; 0)	0 (0; 1)	0	0 (0; 0)	0 (0; 1)	0
Outpatient visits (specialist or nonspecialist)									
Visits, *n*	17,763	36,146	18,383 (103.5)	687	2172	1485 (216.2)	241	1252	1011 (419.5)
Patients, *n*	2865	3106	241 (8.4)	113	145	32 (28.3)	48	65	17 (35.4)
Mean ± SD	5.0 ± 6.7	10.2 ± 13.5	5.2	4.2 ± 6.5	13.2 ± 18.8	9.1	2.9 ± 7.9	15.3 ± 21.7	12.3
Median (Q1; Q3)	3 (1; 7)	6 (2; 13)	3	2 (0; 5.5)	7 (2; 16)	5	1 (0; 3)	7 (2; 15)	6
Emergency department visits									
Visits, *n*	445	595	150 (33.7)	34	51	17 (50.0)	13	16	3 (23.1)
Patients, *n*	356	428	72 (20.2)	27	34	7 (25.9)	9	9	0 (0.0)
Mean ± SD	0.1 ± 0.4	0.2 ± 0.6	0.0	0.2 ± 0.5	0.3 ± 0.7	0.1	0.2 ± 0.5	0.2 ± 0.7	0.0
Median (Q1; Q3)	0 (0; 0)	0 (0; 0)	0	0 (0; 0)	0 (0; 0)	0	0 (0; 0)	0 (0; 0)	0
Prescription classes									
Prescriptions, *n*	5374	6882	1508 (28.1)	235	397	162 (68.9)	104	260	156 (150.0)
Patients, *n*	2128	2373	245 (11.5)	91	112	21 (23.1)	36	56	20 (55.6)
Mean ± SD	1.5 ± 1.8	1.9 ± 2.2	0.4	1.4 ± 1.9	2.4 ± 2.8	1.0	1.3 ± 1.9	3.2 ± 3.6	1.9
Median (Q1; Q3)	1 (0; 2)	1 (0; 3)	0	1 (0; 2)	1 (0; 4)	0	0 (0; 2)	3 (0; 5)	3
Inpatient visits									
Visits, *n*	19	76	57 (300.0)	3	22	19 (633.3)	0	40	40 (NC)
Patients, *n*	18	63	45 (250.0)	3	16	13 (433.3)	0	15	15 (NC)
Mean ± SD	0.0 ± 0.1	0.0 ± 0.2	0.0	0.0 ± 0.1	0.1 ± 0.5	0.1	0.0 ± 0.0	0.5 ± 1.5	0.5
Median (Q1; Q3)	0 (0; 0)	0 (0; 0)	0	0 (0; 0)	0 (0; 0)	0	0 (0; 0)	0 (0; 0)	0
Inpatient lab tests									
Tests, *n*	1	10	9 (900.0)	0	9	9 (NC)	0	13	13 (NC)
Patients, *n*	1	6	5 (500.0)	0	3	3 (NC)	0	2	2 (NC)
Mean ± SD	0.0 ± 0.0	0.0 ± 0.1	0.0	0.0 ± 0.0	0.1 ± 0.4	0.1	0.0 ± 0.0	0.2 ± 1.2	0.2
Median (Q1; Q3)	0 (0; 0)	0 (0; 0)	0	0 (0; 0)	0 (0; 0)	0	0 (0; 0)	0 (0; 0)	0
Length of hospital stay									
Days, *n*	77	388	311 (403.9)	9	162	153 (1700.0)	0	627	627 (NC)
Patients, *n*	18	63	45 (250.0)	3	16	13 (433.3)	0	15	15 (NC)
Mean ± SD	4.3 ± 4.0	6.2 ± 7.3	1.9 (44.0)	3.0 ± 2.0	10.1 ± 10.6	7.1 (237.5)	NC	41.8 ± 54.3	NC
Median (Q1; Q3)	3 (2; 5)	4 (3; 5)	1	3 (1; 5)	5.5 (3.5; 14.0)	2.5	NC	7 (3; 79)	NC
Length of ICU stay									
Days, *n*	1	6	5 (500.0)	0	9	9 (NC)	0	16	16 (NC)
Patients, *n*	1	6	5 (500.0)	0	4	4 (NC)	0	7	7 (NC)
Mean ± SD	0.06 ± 0.24	0.10 ± 0.30	0.04 (71.4)	0.0 ± 0.0	0.6 ± 1.5	0.6 (NC)	NC	1.1 ± 2.1	NC
Median (Q1; Q3)	0 (0; 0)	0 (0; 0)	0	0 (0; 0)	0 (0; 0.5)	0	NC	0 (0; 1)	NC
Patients with invasive mechanical ventilation use, *n* (%)	0 (0.0)	1 (0.0)	1 (NC)	0 (0.0)	1 (0.6)	1 (NC)	0 (0.0)	3 (3.7)	3 (NC)
Patients with noninvasive mechanical ventilation use, *n* (%)	0 (0.0)	1 (0.0)	1 (NC)	0 (0.0)	1 (0.6)	1 (NC)	0 (0.0)	2 (2.4)	2 (NC)
Patients with supplemental oxygen use, *n* (%)	0 (0.0)	1 (0.0)	1 (NC)	0 (0.0)	1 (0.6)	1 (NC)	0 (0.0)	5 (6.1)	5 (NC)
Patients with readmission within 30 days, *n* (%)	0 (0.0)	7 (0.2)	7 (NC)	0 (0.0)	7 (4.3)	7 (NC)	0 (0.0)	9 (11.0)	9 (NC)

*ICU*, intensive care unit; *NC*, not calculable; *Q1*, quartile 1; *Q3*, quartile 3

For visits, tests, prescriptions and procedures, means were calculated as the total value divided by the total number of patients within the cohort

For length of hospital stay and ICU stay, means were calculated as the total number of days divided by the number of patients in the cohort with any inpatient hospital stay

^a^The baseline period was the 12 months before the index date, and the post-acute phase spanned from 1 to 13 months after the index date

Increases in healthcare utilization were also represented by a shift in patterns of discharge status (Table 7). The percentages of hospitalized patients who were discharged to home or self-care were 95.5% during the baseline phase and 65.2% during the post-acute phase. During the post-acute phase, larger percentages of patients were discharged to either a home care service or other facility (such as a hospice or skilled nursing facility), were transferred within the institution, or had an unknown discharge status.

**Table 7:** Hospital discharge status during the baseline and post-acute phases^a^ in the overall population (*N* = 3792)

	**Baseline phase**	**Post-acute phase**
Hospitalizations, *N*	22	138
Discharge status, *n* (%)		
Discharged to home or self-care	21 (95.5)	90 (65.2)
Discharged to home under care of home health service organization	0 (0.0)	5 (3.6)
Discharged to other facility^a^	0 (0.0)	16 (11.6)
Still patient/transferred within institution	0 (0.0)	8 (5.8)
Unknown status	1 (4.5)	19 (13.8)

Percentages were calculated in relation to the total number of hospital discharges during the specified period

^a^Includes short-term general hospital, skilled nursing facility, intermediate care facility, federal healthcare facility, home hospice, medical facility hospice, inpatient rehabilitation facility, long-term care hospital, nursing facility certified under Medicare, psychiatric hospital or psychiatric distinct part/unit of a hospital, critical access hospital or other type of healthcare institution

### Healthcare costs

During the post-acute phase, total medical costs (including prescription, inpatient, and outpatient costs) increased from baseline by 177.9% in the overall population (Table 8). When stratified by disposition during acute COVID-19 illness, cost increases were highest among those admitted to the ICU during acute COVID-19 (+ 1694.6%) but were also apparent among those who were not hospitalized (+ 138.4%) (Table 9). Increases of varying magnitudes were observed across all measures of healthcare costs and across all cohorts.

**Table 8:** Medical costs during the baseline and post-acute phases^a^ in the overall population (*N* = 3792)

Cost description	Baseline phase	Post-acute phase	Change from baseline to post-acute phase, Δ (% change)
**Inpatient visits**			
Total cost	337,095	3,531,778	3,194,684 (947.7)
Standard costs, patient *n*	21	94	
Mean ± SD	16,052 ± 9903	37,572 ± 44,018	21,520 (134.1)
Median (Q1; Q3)	14,335 (11,150; 20,114)	17,503 (11,376; 45,755)	3169 (22.1)
Nonzero costs, patient *n*	21	91	70 (333.3)
Mean ± SD	160,052 ± 9903	38,811 ± 44,199	22,759 (141.8)
Median (Q1; Q3)	14,335 (11,150; 20,114)	17,633 (11,786; 53,778)	3298 (23.0)
**Readmission**			
Total cost	0	1,295,928	1,295,928 (NC)
Standard costs, patient *n*	0	23	
Mean ± SD	NC	56,345 ± 51,072	56,345 (NC)
Median (Q1; Q3)	NC	38,770 (14,209; 100,488)	38,770 (NC)
Nonzero costs, patient *n*	0	22	22 (NC)
Mean ± SD	NC	58,906 ± 50,740	58,906 (NC)
Median (Q1; Q3)	NC	39,325 (16,802; 100,488)	39,325 (NC)
**Outpatient visits**			
Total cost	4,701,415	12,257,210	7,555,795 (160.7)
Standard costs, patient *n*	3026	3316	
Mean ± SD	1554 ± 2609	3696 ± 10,454	2143 (137.9)
Median (Q1; Q3)	664 (278; 1773)	1371 (511; 3369)	707 (106.6)
Nonzero costs, patient *n*	3025	3315	290 (9.6)
Mean ± SD	1554 ± 2609	3698 ± 10,455	2143 (137.9)
Median (Q1; Q3)	664 (278; 1773)	1371 (511; 3372)	707 (106.5)
**Emergency department visits**			
Total cost	778,045	1,088,805	310,760 (39.9)
Standard costs, patient *n*	392	471	
Mean ± SD	1985 ± 1487	2312 ± 2205	327 (16.5)
Median (Q1; Q3)	1638 (1057; 2665)	1687 (1074; 2774)	48 (3.0)
Nonzero costs, patient *n*	391	471	80 (20.5)
Mean ± SD	1990 ± 1485	2312 ± 2205	322 (16.2)
Median (Q1; Q3)	1643 (1061; 2668)	1687 (1074; 2774)	44 (2.7)
**Prescription claims**			
Total cost	1,227,016	1,620,145	393,129 (32.0)
Standard costs, patient *n*	2327	2603	
Mean ± SD	527 ± 2081	622 ± 2212	95 (18.0)
Median (Q1; Q3)	74 (21; 239)	86 (31; 304)	13 (17.6)
Nonzero costs, patient *n*	2327	2603	276 (11.9)
Mean ± SD	527 ± 2081	622 ± 2212	95 (18.0)
Median (Q1; Q3)	74 (21; 239)	86 (31; 304)	13 (17.6)
**All medical costs (outpatient, inpatient, and prescription claims)**			
Total cost	6,265,526	17,409,133	11,143,608 (177.9)
Standard costs, patient *n*	3215	3430	
Mean ± SD	1949 ± 3655	5076 ± 15,425	3127 (160.4)
Median (Q1; Q3)	768 (286; 2068)	1536 (526; 3921)	767 (99.9)
Nonzero costs, patient *n*	3214	3430	216 (6.7)
Mean ± SD	1949 ± 3655	5076 ± 15,425	3126 (160.4)
Median (Q1; Q3)	769 (286; 2068)	1536 (526; 3921)	767 (99.7)

*NC* not calculable, *Q1* quartile 1, *Q3* quartile 3

Standard cost patient *n*’s (used to calculate standard mean and median) reflect the number of patients who had any healthcare encounter for the specified outcome (e.g., all patients with ≥ 1 outpatient visit during the specified period). Nonzero cost patient *n*’s (used to calculate nonzero mean and median) reflect the number of patients who had any costs associated with the specified outcome (e.g., all patients with costs > 0 attributable to outpatient visits)

^a^All costs are in US dollars rounded to the nearest dollar. The baseline phase was the 12 months before the index date, and the post-acute phase spanned from 1 to 13 months after the index date

**Table 9:** Medical costs during the baseline and post-acute phases^a^ stratified by disposition during acute COVID-19

Cost description	No hospitalization (*n* = 3546)			Hospitalization without ICU admission (*n* = 164)			ICU admission (*n* = 82)		
	**Baseline phase**	**Post-acute phase**	**Change from baseline to post-acute phase,** **Δ (% change)**	**Baseline phase**	**Post-acute phase**	**Change from baseline to post-acute phase, Δ (% change)**	**Baseline phase**	**Post-acute phase**	**Change from baseline to post-acute phase, Δ (% change)**
**Inpatient visits**									
Total cost	304,291	2,062,445	1,758,153 (577.8)	32,803	486,273	453,469 (1382.4)	0	983,061	983,061 (NC)
Standard costs, patient *n*	18	63		3	16		0	15	
Mean ± SD	16,905 ± 9847	32,737 ± 38,912	15,832 (93.7)	10,934 ± 10,516	30,392 ± 28,430	19,458 (177.9)	NC	65,537 ± 65,556	65,537 (NC)
Median (Q1; Q3)	14,777 (11,159; 20,114)	16,007 (11,786; 44,054)	1229 (8.3)	10,701 (537; 21,566)	27,602 (2506; 49,195)	16,902 (158.0)	NC	40,266 (4928; 120,540)	40,266 (NC)
Nonzero costs, patient *n*	18	63	45 (250.0)	3	13	10 (333.3)	0	15	15 (NC)
Mean ± SD	16,905 ± 9847	32,737 ± 38,912	15,832 (93.7)	10,934 ± 10,516	37,406 ± 26,946	26,471 (242.1)	NC	65,537 ± 65,556	65,537 (NC)
Median (Q1; Q3)	14,777 (11,159; 20,114)	16,007 (11,786; 44,054)	1229 (8.3)	10,701 (537; 21,566)	38,496 (16,802; 53,778)	27,795 (259.7)	NC	40,266 (4928; 120,540)	40,266 (NC)
**Readmission**									
Total cost	0	367,979	367,979 (NC)	0	236,797	236,797 (NC)	0	691,151	691,151 (NC)
Standard costs, patient *n*	0	7		0	7		0	9	
Mean ± SD	NC	52,568 ± 56,501	52,568 (NC)	NC	33,828 ± 24,414	33,828 (NC)	NC	76,795 ± 58,354	76,795 (NC)
Median (Q1; Q3)	NC	25,493 (12,064; 125,240)	25,493 (NC)	NC	37,651 (16,802; 53,778)	37,651 (NC)	NC	65,694 (38,770; 118,210)	65,694 (NC)
Nonzero costs, patient *n*	0	7	7 (NC)	0	6	6 (NC)	0	9	9 (NC)
Mean ± SD	NC	52,568 ± 56,501	52,568 (NC)	NC	39,466 ± 21,171	39,466 (NC)	NC	76,795 ± 58,354	76,795 (NC)
Median (Q1; Q3)	NC	25,493 (12,064; 125,240)	25,493 (NC)	NC	38,766 (17,217; 53,778)	38,766 (NC)	NC	65,694 (38,770; 118,210)	65,694 (NC)
**Outpatient visits**									
Total cost	4,440,316	10,621,198	6,180,882 (139.2)	186,676	1,180,731	994,056 (532.5)	74,423	455,281	380,857 (511.7)
Standard costs, patient *n*	2865	3106		113	145		48	65	
Mean ± SD	1550 ± 2616	3420 ± 8433	1870 (120.6)	1652 ± 2294	8143 ± 29,601	6491 (392.9)	1550 ± 2910	7004 ± 13,389	5454 (351.7)
Median (Q1; Q3)	667 (280; 1762)	1351 (510; 3300)	684 (102.5)	626 (275; 1962)	1752 (465; 5795)	1126 (180.0)	393 (160; 1229)	1672 (743; 4673)	1279 (325.5)
Nonzero costs, patient *n*	2864	3105	241 (8.4)	113	145	32 (28.3)	48	65	17 (35.4)
Mean ± SD	1550 ± 2617	3421 ± 8434	1870 (120.6)	1652 ± 2294	8143 ± 29,601	6491 (392.9)	1550 ± 2910	7004 ± 13,389	5454 (351.7)
Median (Q1; Q3)	667 (280; 1764)	1351 (510; 3300)	684 (102.5)	626 (275; 1962)	1752 (465; 5795)	1126 (180.0)	393 (160; 1229)	1672 (743; 4673)	1279 (325.5)
**Emergency department visits**									
Total cost	708,502	985,099	276,597 (39.0)	48,565	81,825	33,260 (68.5)	20,978	21,880	903 (4.3)
Standard costs, patient *n*	356	428		27	34		9	9	
Mean ± SD	1990 ± 1472	2302 ± 2198	311 (15.6)	1799 ± 1419	2407 ± 2466	608 (33.8)	2331 ± 2258	2431 ± 1595	100 (4.3)
Median (Q1; Q3)	1661 (1064; 2676)	1686 (1080; 2745)	25 (1.5)	1491 (951; 2391)	1581 (724; 3152)	90 (6.1)	1012 (905; 3223)	1991 (1422; 2774)	978 (96.7)
Nonzero costs, patient *n*	355	428	73 (20.6)	27	34	7 (25.9)	9	9	0 (0.0)
Mean ± SD	1996 ± 1470	2302 ± 2198	306 (15.3)	1799 ± 1419	2407 ± 2466	608 (33.8)	2331 ± 2258	2431 ± 1595	100 (4.3)
Median (Q1; Q3)	1663 (1067; 2684)	1686 (1080; 2745)	22 (1.3)	1491 (951; 2391)	1581 (724; 3152)	90 (6.1)	1012 (905; 3223)	1991 (1422; 2774)	978 (96.7)
**Prescription claims**									
Total cost	1,169,654	1,417,668	248,013 (21.2)	48,249	141,684	93,435 (193.7)	9112	60,793	51,681 (567.2)
Standard costs, patient *n*	2197	2432		94	114		36	57	
Mean ± SD	532 ± 2109	583 ± 2083	51 (9.5)	513 ± 1751	1243 ± 4089	730 (142.1)	253 ± 718	1067 ± 2016	813 (321.4)
Median (Q1; Q3)	76 (22; 243)	84 (31; 291)	8 (10.1)	46 (16; 171)	131 (35; 628)	84 (181.1)	43 (22.9; 148.5)	139 (55; 760)	95 (219.2)
Nonzero costs, patient *n*	2197	2432	235 (10.7)	94	114	20 (21.3)	36	57	21 (58.3)
Mean ± SD	532 ± 2109	583 ± 2083	51 (9.5)	513 ± 1751	1243 ± 4089	730 (142.1)	253 ± 718	1067 ± 2016	813 (321.4)
Median (Q1; Q3)	76 (22; 243)	84 (31; 291)	8 (10.1)	46 (16; 171)	131 (35; 628)	84 (181.1)	43 (22.9; 148.5)	139 (55; 760)	95 (219.2)
**All medical costs (outpatient, inpatient, and prescription claims)**									
Total cost	5,914,262	14,101,310	8,187,048 (138.4)	267,728	1,808,688	1,540,960 (575.6)	83,535	1,499,135	1,415,600 (1694.6)
Standard costs, patient *n*	3034	3211		128	152		53	67	
Mean ± SD	1949 ± 3668	4392 ± 11,640	2442 (125.3)	2092 ± 3625	11,899 ± 35,146	9808 (468.9)	1576 ± 2890	22,375 ± 50,108	20,799 (1319.6)
Median (Q1; Q3)	776 (288; 2068)	1510 (521; 3792)	734 (94.5)	626 (235; 2050)	1990 (439; 7527)	1364 (218.0)	379 (161; 1213)	2264 (940; 9140)	1885 (497.8)
Nonzero costs, patient *n*	3033	3211	178 (5.9)	128	152	24 (18.8)	53	67	14 (26.4)
Mean ± SD	1950 ± 3669	4392 ± 11,640	2442 (125.2)	2092 ± 3625	11,899 ± 35,146	9808 (468.9)	1576 ± 2890	22,375 ± 50,108	20,799 (1319.6)
Median (Q1; Q3)	776 (289; 2068)	1510 (521; 3792)	734 (94.5)	626 (235; 2050)	1990 (439; 7527)	1364 (218.0)	379 (161; 1213)	2264 (940; 9140)	1885 (497.8)

*ICU* intensive care unit, *NC* not calculable, *Q1* quartile 1, *Q3* quartile 3

Standard cost patient *n*’s (used to calculate standard mean and median) reflect the number of patients who had any healthcare encounter for the specified outcome (e.g., all patients with ≥ 1 outpatient visit during the specified period). Nonzero cost patient *n*’s (used to calculate nonzero mean and median) reflect the number of patients who had any costs associated with the specified outcome (e.g., all patients with costs > 0 attributable to outpatient visits)

^a^All costs are in US dollars rounded to the nearest dollar. The baseline phase was the 12 months before the index date, and the post-acute phase spanned from 1 to 13 months after the index date

Matching the trends in healthcare utilization (Table 5), the greatest cost increases were associated with inpatient hospitalizations, including readmissions within 30 days of hospital discharge (Table 8). Smaller but substantial increases were associated with outpatient visits, including ED visits, and prescription claims. With the exception of ED visit costs, all cost increases were greatest among those admitted to the ICU during acute COVID-19 (Table 9).

## Discussion

This retrospective analysis of adults aged < 65 years without any underlying high-risk conditions identified increases in diagnoses, medical prescriptions, healthcare utilization events, and associated costs during the year after the acute phase of COVID-19. As observed previously [10] and in our companion manuscript regarding high-risk patients [20], those who were hospitalized with or without ICU admission during the acute phase had the greatest increase in comorbidities and healthcare resource utilization burden. However, the burden was apparent across all 3 cohorts, including those who were not hospitalized for COVID-19.

Many of the changes observed in this cohort were similar to observations among high-risk patients [20]. Most notably, the greatest increase in both studies was in the percentages of patients with blood-related diseases, which has been identified previously as a feature of post-COVID conditions [21]. In the present study, new diagnoses of blood disorders increased by > 150% during the post-acute phase compared with baseline, even among the cohort of patients who were not hospitalized during the acute phase of illness. New diagnoses of neurological and psychiatric diseases also increased in the year following acute COVID-19 in both patients with and without any risk factors for severe COVID-19, regardless of the level of care setting during the acute phase of illness, which is consistent with previous reports of long-term COVID-19 sequelae [22, 23]. We observed a 27% decrease in respiratory disease during the post-acute phase; similar to results from the high-risk population, this decrease reflected a large increase in chronic lower respiratory diseases that was outweighed by a combination of smaller decreases in acute upper and lower respiratory infections, influenza, and pneumonia. Distinct from observations in high-risk patients, analysis of the cohort described here also revealed a 123% increase in endocrine, nutritional, and metabolic diseases and a 76% increase in diseases of the digestive system.

The analysis of medication use demonstrated the greatest increase in hormone prescriptions, including primarily injectable, oral, and topical glucocorticoids and corticoids (e.g., prednisone, methylprednisolone, and dexamethasone). These medications were typically not prescribed during the baseline phase and were likely being used for the treatment of persistent COVID-19 symptoms, such as joint stiffness and muscle pain [24]. Increases in hormone use were followed by vascular and musculoskeletal agents, and increases across the 3 classes of medications were observed across all levels of care during acute COVID-19. Those results were unique to this population and not observed among high-risk patients [20]. However, when the analysis was conducted including medication classes prescribed to < 2% of the baseline population, a 277% increase in hemostatic modifier prescriptions and a 106% increase in blood factor prescriptions were identified, consistent with results of the same analysis among high-risk patients. It is logical that several of the medication classes prescribed to ≥ 2% of the baseline population in the high-risk patient cohort were prescribed to < 2% of the baseline population in the cohort evaluated here because these patients were overall younger and healthier at baseline.

Healthcare resource utilization and costs were higher during the post-acute phase compared with the baseline phase for all evaluated measures, including outpatient visits, ED visits, inpatient visits and LOS, ICU admission and LOS, 30-day readmissions, and prescriptions. The likelihood of patients being discharged to another hospital department or long-term care facility also increased. The most striking cost changes were observed across measures related to inpatient resources, including an overall increase in inpatient visit costs of nearly 950% in the post-acute phase and resulting in a 178% increase in total medical costs in the overall population.

Cost increases varied in magnitude but were observed for every healthcare outcome across all categories of the acute COVID-19 level of care. Even among those who were not hospitalized during the acute phase of COVID-19, inpatient visit costs during the post-acute phase increased by 578%, outpatient visit costs increased by 139%, and total medical costs increased by 138%. This trend was similar to observations made in the cohort of high-risk patients [20] but was a more surprising result given that the patients in the cohort described here were aged < 65 years and lacked any comorbid conditions placing them at risk of severe COVID-19.

Taken together, health and healthcare resource use results suggest that the risk factors associated with developing post-COVID conditions may be distinct from the risk factors that predict severity of acute disease. Similarly to how biomarkers have been characterized to predict the course of acute COVID-19 [25], there may be a unique set of biomarkers associated with the development of long-term adverse health outcomes. One recent study identified biomarkers associated with vascular transformation among long-COVID patients [26], which is consistent with findings in our present study and companion report regarding a high prevalence of blood-related diseases in the post-acute phase. Importantly, regardless of the mechanisms involved, results indicate that the health and economic impacts of COVID-19 may extend beyond the acute phase of illness even among the wide swath of the population that is relatively young and healthy and has mild symptoms upon infection.

Although limited data are available on strategies to reduce the risk of post-COVID conditions, vaccination against COVID-19 appears to be protective. In a recent prospective study from Antonelli and colleagues [27], fully vaccinated adults with breakthrough infections were less likely than unvaccinated controls to experience symptoms of COVID-19 lasting ≥ 28 days; the effect was observed among both older adults and adults aged < 60 years. In a retrospective cohort study from Taquet and colleagues [28], vaccination among adults who contracted COVID-19 was associated with steep reductions in risk of several long-term adverse health outcomes. Although authors of the Taquet study did not find COVID-19 vaccination to be associated with reduced risk of what they termed “long COVID features” (a collection of specific abdominal, respiratory, psychiatric, and pain-related symptoms), they did identify significantly lower risk of many of the diagnoses discussed in the present report, including blood disorders, muscle disease, certain neurological conditions, and chronic respiratory conditions. Because these outcomes were measured over a 6-month period after infection, they included diagnoses during both the acute and post-acute phases. Highlighting the importance of vaccination even among those not in a high-risk group, protective effects of vaccination against many post-acute sequelae were most robust among individuals aged < 60 years.

Recently, emerging data have also suggested that antiviral treatment for COVID-19, such as nirmatrelvir/ritonavir (Paxlovid®, Pfizer Inc, New York, NY, USA), may reduce the likelihood of developing PASC. In a large study of patients from the Veterans Affairs database [29], authors found that individuals prescribed nirmatrelvir-ritonavir during the acute phase of COVID-19 had reduced risks of prespecified sequelae (including cardiovascular, hematologic, and neurologic disorders), post-acute hospitalization, and post-acute death, regardless of vaccination status. A small case series has furthermore suggested that nirmatrelvir administered during the post-acute phase may alleviate long-term symptom burden [30], although more systematic study of these effects is warranted before conclusions can be drawn.

Our study had several strengths. Primarily, having all patients serve as their own control inherently adjusted for potential confounders, such as demographics and health-seeking behavior. To our knowledge, this was also the first study that evaluated the economic impact of post-acute COVID-19 in adults who were < 65 years of age and had no underlying comorbidities placing them at risk of severe acute COVID-19. The ability to pair these broad, descriptive data with the identical analysis in a cohort of adults with high-risk conditions [20] is of significant value in understanding differences between characteristics that predict acute versus post-acute COVID-19 outcomes. A limitation of our study was that the cohort was confined to individuals with commercial insurance; all enrolled participants were also diagnosed early in the pandemic and survived the acute phase of COVID-19. Additionally, there was no method of confirming that any adverse health outcomes reported here were related to COVID-19. There was also a possibility for incomplete data capture owing to nonbillable diagnoses, and our results may have been influenced by surveillance bias, whereby contracting COVID-19 led to higher medical scrutiny following diagnosis. Finally, the study was conducted in a period before vaccination and previous SARS-CoV-2 infection and before the emergence of SARs-CoV-2 variants of concern. Such factors could limit the generalizability of the findings to the present. Baseline assessments were also performed in the prepandemic period; whereas post-acute COVID-19 assessments were performed during a public health emergency in which healthcare practices could have changed. Thus, clinical burden and health costs during the post-acute COVID-19 period may have been impacted by altered heathcare practices.

## Conclusion

Our data suggest that the health and economic burden of COVID-19 stretches well beyond the acute phase of illness, even among younger individuals without preexisting conditions whose acute infection did not merit hospitalization. Understanding the nature and extent of post-COVID conditions, as well as the unique populations at risk, is critical to evaluating the true societal cost–benefit of interventions such as COVID-19 vaccination and treatment.

### Supplementary Information

**Additional file 1: Table S1.** Reasons for Exclusion From the Study. *ICD-10* International Classification of Diseases, 10th Revision, *LTCF *long-term care facility, *SNF *skilled nursing facility. ^a^There is no accurate date of death available, only month and year of death, so it was approximated to the last day of the month.

**Additional file 2: Table S2.** Hormone Prescriptions During the Baseline and Post-Acute Phase^a^ in the Overall Population (*N*=3792)^b^. *ACTH* adrenocorticotropic hormone, *NC* not calculable, *USC* Uniform System of Classification. ^a^The baseline period was the 12 months before the index date, and the post-acute phase spanned from 1 to 13 months after the index date. ^b^Includes all prescriptions, including those prescribed to <2% of the baseline population (which were excluded from the main analysis).

**Additional file 3: Table S3.** New Medication Prescriptions With a ≥100% Increase From the Baseline to the Post-Acute Phase^a^ in the Overall Population (*N*=3792)^b^. *USC* Uniform System of Classification. ^a^The baseline period was the 12 months before the index date, and the post-acute phase spanned from 1 to 13 months after the index date. ^b^Includes all prescriptions, including those prescribed to <2% of the baseline population (which were excluded from the main analysis).

## Data Availability

Upon request, and subject to review, Pfizer will provide the data that support the findings of this study. Subject to certain criteria, conditions, and exceptions, Pfizer may also provide access to the related individual de-identified participant data. See https://www.pfizer.com/science/clinical-trials/trial-data-and-results for more information.

## Abbreviations

ACTH: Adrenocorticotropic hormone
CDC: US Centers for Disease Control and Prevention
CDM: Optum’s de-identified Clinformatics® Data Mart Database
CPT: Current Procedural Terminology
ED: Emergency department
HCPCS: Healthcare Common Procedure Coding System
ICD-10: International Classification of Diseases, 10th Revision
ICD-10-CM: International Classification of Diseases, 10th Revision, Clinical Modification
ICD-10-PCS: International Classification of Diseases, 10th Revision, Procedure Coding System
ICU: Intensive care unit
LOS: Length of stay
LTCF: Long-term care facility
NC: Not calculable
NDC: National Drug Code
PASC: Post-acute sequelae of COVID-19
Q1: Quartile 1
Q3: Quartile 3
SAS: Statistical analysis software
SNF: Skilled nursing facility
USC: Uniform System of Classification
USD: United States dollars
