# Supplementary material for: Substantial health and economic burden of COVID-19 during the year after acute illness among US adults not at high risk of severe COVID-19
Source: BMC Med. 2024 Feb 2;22:47. doi: 10.1186/s12916-023-03235-5 (PMC10835856; doi:10.1186/s12916-023-03235-5)
Supplement: Supplementary file 1 — Additional file 1: Table S1. Reasons for Exclusion From the Study. ICD-10 International Classification of Diseases, 10th Revision, LTCF long-term care facility, SNF skilled nursing facility. aThere is no accurate date of death available, only month and year of death, so it was approximated to the last day of the month. [file 12916_2023_3235_MOESM1_ESM.pdf]

Additional Files

Table S1. Reasons for Exclusion From the Study

Cohort Selection Step	N	%
All patients enrolled in database during April 2020–May 2020	16,909,650	100
and with ≥1 ICD-10 diagnosis of confirmed COVID-19 in any position	70,924	0.419
and exclude patients with an ICD-10 code for confirmed COVID-19 (U07.1) before April 1, 2020 (preindex)	68,297	0.404
and with continuous enrollment 12 months (360 days) before the index date (45-day gap)	50,463	0.298
and with continuous enrollment 13 months (390 days) after the index date (45-day gap)	32,551	0.192
and aged ≥18 at the index date	32,054	0.190
and without any LTCF/SNF/inpatient rehab/hospice claim before or at the index date	23,458	0.139
and exclude patients admitted earlier than 5 days before IP index COVID-19 diagnosis	23,392	0.138
and alive in the data/no death[a] code at 30 days after the COVID-19 diagnosis date (index date)	23,350	0.138
and does NOT have ≥1 characteristic or condition (within 12 months before COVID-19 diagnosis - excluding index date) associated with higher risk for severe COVID-19	3,792	0.022

ICD-10: International Classification of Diseases, 10th Revision; LTCF=long-term care facility, SNF=skilled nursing facility.

[a]There is no accurate date of death available, only month and year of death, so it was approximated to the last day of the month.
