# Supplementary material for: Substantial health and economic burden of COVID-19 during the year after acute illness among US adults not at high risk of severe COVID-19
Source: BMC Med. 2024 Feb 2;22:47. doi: 10.1186/s12916-023-03235-5 (PMC10835856; doi:10.1186/s12916-023-03235-5)
Supplement: Supplementary file 2 — Additional file 2: Table S2. Hormone Prescriptions During the Baseline and Post-Acute Phasea in the Overall Population (N=3792)b. ACTH adrenocorticotropic hormone, NC not calculable, USC Uniform System of Classification. aThe baseline period was the 12 months before the index date, and the post-acute phase spanned from 1 to 13 months after the index date. bIncludes all prescriptions, including those prescribed to <2% of the baseline population (which were excluded from the main analysis). [file 12916_2023_3235_MOESM2_ESM.pdf]

Table S2. Hormone Prescriptions During the Baseline and Post-Acute Phase[a] in the Overall Population (N=3792)[b]

USC Hormone Class Description	Baseline Phase, n (%)	Post-Acute Phase, n (%)	Change From Baseline to Post-Acute Phase, Δ (% Change)
Glucocorticoids plain oral	0 (0.0)	366 (9.7)	366 (NC)
Glucocorticoid plain injectable	62 (1.6)	225 (5.9)	163 (262.9)
Corticoids plain dermatological	145 (3.8)	220 (5.8)	75 (51.7)
Progestogen injectable	1 (0.0)	4 (0.1)	3 (300.0)
Other ACTH & mineralocorticoids	1 (0.0)	3 (0.1)	2 (200.0)
Progestogen oral	34 (0.9)	36 (0.9)	2 (5.9)
Androgens injectable	11 (0.3)	13 (0.3)	2 (18.2)
Progesterone antagonists	0 (0.0)	1 (0.0)	1 (NC)
Estrogens injectable	0 (0.0)	1 (0.0)	1 (NC)
Glucocorticoids plain other	3 (0.1)	3 (0.1)	0 (0.0)
Progestogen vaginal	2 (0.1)	2 (0.1)	0 (0.0)
Gonadotrophins	5 (0.1)	4 (0.1)	–1 (–20.0)
Androgens transdermal	3 (0.1)	2 (0.1)	–1 (–33.3)
Corticoid combs dermatological	2 (0.1)	1 (0.0)	–1 (–50.0)

ACTH, adrenocorticotropic hormone; NC, not calculable; USC, Uniform System of Classification.
[a]The baseline period was the 12 months before the index date, and the post-acute phase spanned from 1 to 13 months after the index date.
[b]Includes all prescriptions, including those prescribed to <2% of the baseline population (which were excluded from the main analysis).
