# Supplementary material for: Substantial health and economic burden of COVID-19 during the year after acute illness among US adults not at high risk of severe COVID-19
Source: BMC Med. 2024 Feb 2;22:47. doi: 10.1186/s12916-023-03235-5 (PMC10835856; doi:10.1186/s12916-023-03235-5)
Supplement: Supplementary file 3 — Additional file 3: Table S3. New Medication Prescriptions With a ≥100% Increase From the Baseline to the Post-Acute Phasea in the Overall Population (N=3792)b. USC Uniform System of Classification. aThe baseline period was the 12 months before the index date, and the post-acute phase spanned from 1 to 13 months after the index date. bIncludes all prescriptions, including those prescribed to <2% of the baseline population (which were excluded from the main analysis). [file 12916_2023_3235_MOESM3_ESM.pdf]

Table S3. New Medication Prescriptions With a ≥100% Increase From the Baseline to the Post-Acute Phase[a] in the Overall Population (N=3792)[b]

USC Medication Class Description	Baseline Phase, n (%)	Post-Acute Phase, n (%)	Change From Baseline to Post-Acute Phase, Δ (% Change)
Parasympathetic drugs	1 (0.0)	10 (0.3)	9 (900.0)
Tuberculosis therapy	1 (0.0)	5 (0.1)	4 (400.0)
Anthelmintics	3 (0.1)	13 (0.3)	10 (333.3)
Miscellaneous preparations	12 (0.3)	47 (1.2)	35 (291.7)
Hemostatic modifiers	17 (0.4)	64 (1.7)	47 (276.5)
Immunologic agents	2 (0.1)	7 (0.2)	5 (250.0)
Laxatives	34 (0.9)	112 (3.0)	78 (229.4)
Diagnostic aids	66 (1.7)	203 (5.4)	137 (207.6)
Anesthetics	57 (1.5)	165 (4.4)	108 (189.5)
Hormones	228 (6.0)	658 (17.4)	430 (188.6)
Diabetes therapy	38 (1.0)	107 (2.8)	69 (181.6)
Hospital solutions	56 (1.5)	154 (4.1)	98 (175.0)
Cardiac agents	18 (0.5)	47 (1.2)	29 (161.1)
Vascular agents	130 (3.4)	277 (7.3)	147 (113.1)
Blood factors	17 (0.4)	35 (0.9)	18 (105.9)
Nutrients & supplements	13 (0.3)	26 (0.7)	13 (100.0)

USC, Uniform System of Classification.
[a]The baseline period was the 12 months before the index date, and the post-acute phase spanned from 1 to 13 months after the index date.
[b]Includes all prescriptions, including those prescribed to <2% of the baseline population (which were excluded from the main analysis).
